# Recent Advances and Opportunities in Research on Lupus: Environmental Influences and Mechanisms of Disease

**DOI:** 10.1289/ehp.11092

**Published:** 2008-03-05

**Authors:** Glinda S. Cooper, Kathleen M. Gilbert, Eric L. Greidinger, Judith A. James, Jean C. Pfau, Leslie Reinlib, Bruce C. Richardson, Noel R. Rose

**Affiliations:** 1 National Center for Environmental Assessment, U.S. Environmental Protection Agency, Washington, DC, USA; 2 Department of Microbiology and Immunology, University of Arkansas for Medical Sciences/Arkansas Children’s Hospital Research Institute, Little Rock, Arkansas, USA; 3 Division of Rheumatology, University of Miami Miller School of Medicine, Miami Department of Veterans Affairs Medical Center, Miami, Florida, USA; 4 Arthritis and Immunology Program, Oklahoma Medical Research Foundation, and Department of Medicine, University of Oklahoma Health Sciences Center, Oklahoma City, Oklahoma, USA; 5 Center for Environmental Health Sciences, Department of Biomedical and Pharmaceutical Sciences, University of Montana, Missoula, Montana, USA; 6 Division of Extramural Research and Training, National Institute of Environmental Health Sciences, National Institutes of Health, Department of Health and Human Services, Research Triangle Park, North Carolina, USA; 7 University of Michigan and Ann Arbor Veterans Affairs Hospital, Ann Arbor, Michigan, USA; 8 Department of Pathology, Johns Hopkins Center for Autoimmune Disease Research, Johns Hopkins University, Baltimore, Maryland, USA

**Keywords:** adjuvant effect, apoptosis, autoimmune diseases, bystander effect, demethylation, epigenetics, Epstein-Barr virus, silica, systemic lupus erythematosus, trichloroethylene

## Abstract

**Objectives:**

In this review we summarize research on mechanisms through which environmental agents may affect the pathogenesis of lupus, discuss three exposures that have been the focus of research in this area, and propose recommendations for new research initiatives.

**Data sources and synthesis:**

We examined studies pertaining to key mechanistic events and specific exposures. Apoptosis leading to increased production or decreased clearance of immunogenic intracellular self-antigens and defective apoptosis of autoreactive immune cells both have been implicated in the loss of self-tolerance. The adjuvant or bystander effect is also needed to produce a sustained autoimmune response. Activation of toll-like receptors is one mechanism through which these effects may occur. Abnormal DNA methylation may also contribute to the pathogenesis of lupus. Each of the specific exposures we examined—Epstein-Barr virus, silica, and trichloroethylene—has been shown, in humans or in mice, to act upon one or more of these pathogenic steps. Specific recommendations for the continued advancement of our understanding of environmental influences on lupus and other autoimmune diseases include the development and use of mouse models with varying degrees of penetrance and manifestations of disease, identification of molecular or physiologic targets of specific exposures, development and use of improved exposure assessment methodologies, and multisite collaborations designed to examine understudied environmental exposures in humans.

**Conclusions:**

The advances made in the past decade concerning our understanding of mechanisms involved in the development of lupus and the influence of environmental agents on this process provide a strong foundation for further developments in this field.

Systemic lupus erythematosus (SLE, or lupus) is a chronic autoimmune rheumatic disease that may involve serious renal, cardiovascular, and neurologic complications. The pathogenesis of lupus, as well as other autoimmune diseases such as systemic sclerosis and rheumatoid arthritis, is thought to involve complex interactions of multiple genes and environmental agents. In this review we summarize recent research pertaining to potential pathogenic mechanisms of environmental exposures that may be involved in the development of SLE in humans and recommendations for new research to better understand environmental influences and gene–environment interactions in lupus.

## Mechanisms Involved in the Pathogenesis of Lupus

**Figure 1:**
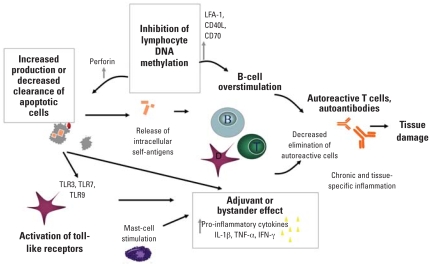
Mechanisms involved in the loss of self-tolerance and development of autoimmune pathology. Activation of innate immune effectors as a danger signal, adjuvant effects, and a decreased clearance of autoreactive cells produce a sustained pathogenic response to the self-antigens that may result from apoptotic debris. Abnormal DNA methylation may also result in increased production and decreased clearance of apoptotic cells through macrophage apoptosis, and overstimulation of B cells.

Recent research has advanced our understanding of mechanisms involved in the loss of tolerance and development of a chronic state of inflammation, with work involving the role of apoptosis in the generation and clearance of immunogenic intracellular self-antigens, adjuvant or bystander effects, toll-like receptors (TLRs) and innate immunity, and abnormal DNA methylation. Environmental exposures may act as an initiating event and influence at other points in the pathogenesis of an autoimmune disease (Figure 1).

### Apoptosis as a source of self-antigens

Immune responses against self-antigens are fundamental to lupus pathogenesis, and dead and dying cells are a major source of the self-antigens targeted in lupus (Walport 2000). Consistent with this idea, immunization of test animals with preparations of dead cells, or preparations expressing epitopes found on dead cells, can induce lupuslike immunity and clinical manifestations (Mevorach et al. 1998). Moreover, mutations that lead to impaired nonimmune clearance of dead cell debris have been shown to be strong risk factors for the development of lupus in animal models (Cohen et al. 2002). Experimental evidence shows that the clearance of dead cells can be overwhelmed when encountering extremely high rates of cell death, leading to the induction of lupuslike disease (Grader-Beck et al. 2007). Some environmental agents have been shown to induce periods of increased cell death (e.g., ultraviolet light, viral infections) (Kuhn et al. 2006). Notably, ultraviolet-B exposure and viral infections can also lead to the production of novel forms of autoantigens [such as ultraviolet radiation–induced, covalently linked protein–RNA conjugates (Andrade et al. 2005)] that may be particularly favorable for the induction of autoimmunity. It is also plausible that environmental exposures may lead to periods of defective or depleted dead cell clearance mechanisms (caused by, for example, complement consumption or reduced local macrophage levels).

### Impaired elimination of autoreactive cells via apoptosis

Although dead cell debris promotes autoreactivity, the process of programmed cell death is also critical to preventing and limiting an autoimmune response. Specifically, apoptosis of immune cells participating in autoimmune responses (e.g., T cells, B cells, dendritic cells) is a major mechanism for the induction and maintenance of self-tolerance (Chen et al. 2006; Stranges et al. 2007). Just as gene mutations leading to defects in immune cell apoptosis have been linked to increased risk for the development of lupus (Drappa et al. 1993), environmental factors influencing the set point at which autoreactive cells undergo apoptosis also appear to influence lupus susceptibility. An example in mice is the ability of estrogen to protect autoreactive B cells from programmed cell death (Venkatesh et al. 2006). Conversely, impairments in the ability of cytotoxic cells to eliminate autoreactive immune cells appear to enhance the risk of lupus (Graham et al. 2005). Environmental exposures that selectively impair apoptosis of immune system cells may predispose to lupus in a manner that synergizes with exposures that increase target tissue apoptosis. Once established, the development of autoreactive immune memory cells (with impaired apoptosis compared with naïve cells) and the induction of lupus target organ injury (with increased local cell death due to the effects of lupus) can lead to a self-sustaining and self-amplifying cycle of lupuslike autoimmunity.

### The adjuvant or bystander effect

What mechanisms prevent the unlimited proliferation of T cells that are capable of recognizing self-antigens? T-cell receptor engagement alone is ineffective in activating the pathways needed to produce the proinflammatory cytokines and other growth factors required for the induction of the pathogenic autoimmune response. A second, non-antigen-specific signal determines whether an encounter with a potential self-antigen is aborted at an early stage, proceeds only to a limited, harmless autoimmune response, or progresses to a pathogenic outcome. The secondary signals involved in modulating the immune response have collectively been termed the “adjuvant effect”; another term for these signals is the “bystander effect” (Rose and Afanasyeva 2003).

Information has accumulated rapidly in recent years that allows us to better understand the adjuvant effect at a molecular level. A vivid illustration of the importance of the adjuvant effect in the induction of autoimmune disease was revealed by studies of autoimmune myocarditis. This disease can be induced in susceptible strains of mice by infection by Coxsackievirus B3 or, alternatively, by immunization with purified cardiac myosin (Hill et al. 2002). The cardiac myosin immunization must, however, be accompanied by a powerful adjuvant, complete Freund adjuvant, which includes the mycobacterium component. Incomplete Freund adjuvant results in the production of myosin-specific antibodies without the occurrence of inflammatory lesions in the heart muscle. In resistant strains of mice that do not develop disease after Coxsackievirus B3 infection or cardiac myosin immunization, cotreatment with bacterial lipopolysaccaride results in a florid disease (Lane et al. 1991). This disease is dependent upon the prompt production of the key early proinflammatory cytokines, interleukin-1 and tumor necrosis factor-α (Lane et al. 1992). Mast cells are critical players in the initiation of the adjuvant effect that occurs early after viral infection (Fairweather et al. 2004).

Another series of experiments used a surrogate marker of the adjuvant effect: a sudden drop in the thyroid hormone thyroxine that occurs after immunization using complete Freund adjuvant (Rocchi et al. 2007). These studies indicate that TLRs or similar receptors of the innate immune response are critical for mounting the adjuvant effect. Thus, active infection or products of the infection can provide the adjuvant effect necessary for the induction of many autoimmune disorders. The adjuvant effect depends upon early non-antigen-specific signals that initiate an innate immune response. It shapes the later adaptive response that is directly responsible for pathogenic autoimmunity. The potential role of other environmental agents in the induction of an adjuvant effect of this type and the role of an adjuvant effect specifically in lupus are important areas of research.

In addition to providing antigenic stimuli to B cells and T cells, nucleic acid components associated with dead cell debris have been shown to activate the adjuvant effect through TLR activation (Hoffman et al. 2004). RNA activation of TLR7 on B cells and plasmacytoid dendritic cells has been particularly strongly associated with lupus pathogenesis, based on its role as the genetic risk gene for a murine model of inherited lupus (Pisitkun et al. 2006) and its ability to induce lupus-associated type I interferon production (Vollmer et al. 2005). The RNA-sensing TLR3 has also been implicated as an inducer of inflammation in a variant form of lupuslike disease (Greidinger et al. 2006), and the DNA-sensing TLR9 has been associated with lupus nephritis in some models (Pawar et al. 2006). However, these innate immune receptors have also been associated with down-regulation of immune responses under some circumstances (Christensen et al. 2006). Adjuvant effect signals may influence the tissues targeted by autoantigen-specific innate immune responses by selectively increasing inflammatory responses in some tissues and decreasing the inflammatory responses in others (Greidinger et al. 2007).

Environmental factors could influence TLR responses and hence the adjuvant effect in lupus in multiple ways. Radiation, chemical toxins, or microbial products may selectively activate or inhibit general innate immune response pathways or selectively influence a single TLR pathway to influence the induction of lupus. Moreover, recent identification of lupus risk factor genes with functions in interferon responses (Graham et al. 2007) and as more general mediators of innate immune signaling, as in the case of STAT4 (Remmers et al. 2007), suggests that innate immune pathways subject to environmental influences in addition to TLRs may prove to be relevant to lupus pathogenesis. Deficiencies in the complement system may result in the aberrant clearance of apoptotic cells, and specific deficiencies have been implicated in the pathogenesis of SLE (Trouw et al. 2008).

### Epigenetics: DNA demethylation and pathogenic T and B cells

Environmental factors may also induce autoimmunity through epigenetic mechanisms. Epigenetics is defined as heritable changes in gene expression that occur without a change in DNA sequence, and the best-characterized mechanism is DNA methylation. DNA methylation, the postsynthetic methylation of cytosines in CG pairs, silences genes by altering chromatin structure into a transcriptionally repressive configuration. The methylation of previously unmethylated sequences is mediated by the *de novo* DNA methyltransferases Dnmt3a and Dnmt3b. A more detailed discussion of methylation and epigenetics is provided in a recent review (Richardson 2007).

DNA methylation patterns are established during development and suppress genes that are inappropriate or detrimental to the function of any given cell type. Inhibiting lymphocyte DNA methylation during mitosis alters gene expression, resulting in immunogenic changes that can alter the response to self-antigens, including overexpression of the adhesion molecule LFA-1 (CD11a/CD18), the cytotoxic molecule perforin, and B-cell costimulatory molecules CD70 and CD40L (Ballestar et al. 2006). LFA-1 overexpressing T cells are autoreactive, resembling the T cells that cause lupuslike chronic graft-versus-host disease. Perforin overexpression contributes to autologous macrophage killing by the autoreactive cells, with subsequent release and impaired clearance of antigenic nucleosomes, whereas CD70 and CD40L overexpression overstimulate B-cell immunoglobulin production (Ballestar et al. 2006; Lu Q et al. 2007; Richardson 2007). The CD40L overexpression occurs only in women, as CD40L is encoded on the X chromosome (Lu Q et al. 2007). Injecting demethylated T cells into genetically identical mice causes a lupuslike disease with anti-DNA antibodies and an immune complex glomerulonephritis (Richardson 2007; Yung et al. 2001). These functional changes suggest a model in which hypomethylated T cells kill macrophages and perhaps other antigen-presenting cells (APCs), causing an increase in apoptotic material that promotes an anti-DNA response, which is further augmented by increased antibody production.

Studies in lupus patients also suggest that T-cell DNA demethylation may be fundamental to the disease process. CD4^+^ T cells from patients with active lupus overexpress LFA-1, perforin, CD70, and CD40L because of demethylation of the same sequences demethylated by a Dnmt inhibitor (Deng et al. 2001). Further, CD4^+^ lupus T cells demonstrate functional changes identical to T cells demethylated *in vitro*, with perforin-dependent autoreactive macrophage killing and CD70/CD40L-dependent B-cell overstimulation (Ballestar et al. 2006; Lu Q et al. 2007; Richardson 2007).

The evidence summarized above suggests that aberrant T-cell DNA methylation contributes to lupus pathogenesis. DNA methylation silences one X chromosome in women, and suppresses parasitic viral DNA. Thus DNA methylation may represent one pathway that may influence the marked sex difference in incidence of lupus in humans, as seen with the example of sex-specific CD40L overexpression and B-cell stimulation. Further support for the association between T-cell DNA methylation and lupus can be found in reports that hydralazine and procainamide, which cause antinuclear antibodies in most people and a lupuslike disease in a subset, are DNA methylation inhibitors. Procainamide acts through inhibition of the DNA methyl transferase enzyme Dnmt1 (Lee et al. 2005), and hydralazine acts through the extracellular signal-regulated kinase (ERK) signaling pathway (Deng et al. 2003). The signaling defect in lupus and in hydralazine-treated T cells maps to protein kinase C (PKC)-δ (Gorelik et al. 2007), supporting commonality of mechanisms. Ultraviolet light also triggers lupus flares, inhibits ERK pathway signaling, and is a DNA methylation inhibitor (Richardson 2007). These examples suggest that other xenobiotics could contribute to the development or exacerbation of lupus in some patients by similar mechanisms. Agents (e.g., dietary deficiencies) that deplete the pool or biosynthesis of the methyl donor or that inhibit key enzymes or the signaling pathways (e.g., increased homocysteine levels) could lead to increased inhibition of the reaction. Finally, reports that methyl donor restriction during fetal development can cause lifelong effects (Waterland and Jirtle 2004) raises the possibility that nutritional deficiencies or exposure to toxicants during pregnancy may affect lupus susceptibility later in life.

## Viral, Solvent, and Particulate Exposures and the Pathogenesis of Autoimmune Disease

In the previous section we described mechanisms through which different environmental exposures could affect the development of lupus and other systemic autoimmune diseases. Here we discuss three examples of exposures. Although consensus does not exist regarding all of the issues with respect to each exposure and the development of lupus, these three diverse exposures have generated interest from lupus researchers, based on potential connections to elements identified in the lupus disease pathway. Of these, the environmental exposure with the most developed literature suggesting a link with SLE is Epstein-Barr virus (EBV). EBV is a nearly ubiquitous pathogen that has been associated with lupus in studies using serologic and DNA measures. Its high worldwide prevalence raises the key question of how a common environmental exposure leads to disease in only a very small subset of infected individuals (Thompson and Kurzrock 2004) and suggests that differences in EBV infection or differences in the host immune response to the infection may be key considerations. Although the first study of occupational respirable silica exposure and systemic autoimmune disease was published almost 100 years ago [reviewed by Brown et al. (2004b); Parks et al. (1999)], it is only in the past decade that this relationship has become the focus of mechanistic studies in animal models, taking its historical label as an adjuvant to the molecular level. These examples further illustrate how exposures can lead to a series of events from the initial initiating impact to multiple levels of the immune response. Trichloroethylene is a commonly used solvent and potential air and water contaminant; solvents have been most consistently associated in epidemiologic studies with systemic sclerosis (Cooper et al. 2002). In experimental studies of trichloroethylene using the MRL+/+ mouse model, however, autoimmune hepatitis, skin inflammation, and alopecia were seen. These observations highlight the critical gaps in our understanding of the expression of autoimmune disease in animals and humans.

### EBV: a summary of three decades of research

**Figure 2:**
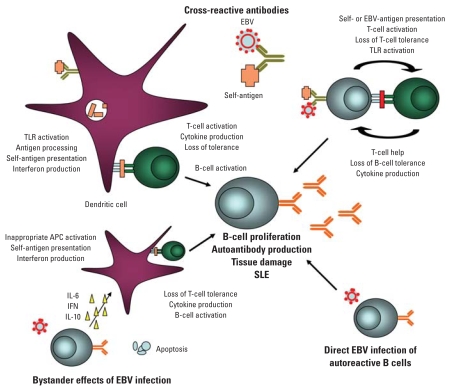
Putative roles for EBV in the initiation and propagation of SLE. Supportive evidence is available for direct effects (e.g., molecular mimicry with production cross-reactive antibodies) and indirect effects (e.g., bystander effects, inappropriate cytokine production, or gene expression).

EBV was offered as a potential environmental factor in SLE as early as 1972. Pediatric lupus patients, used as controls in a study of EBV and childhood lymphoma, were found to have an enriched frequency of EBV serology compared with other children (Dalldorf et al. 1972). During the following few decades, debate raged [for a historical review, see McClain et al. (2001)]. Evidence of induced models, molecular mimicry, adjuvant or bystander effects, viral DNA association, increased viral loads, differential EBV gene expression, and abnormal EBV T-cell and B-cell responses in lupus have recently all provided additional support for potential roles for EBV in lupus [reviewed by Poole et al. (2006)] (Figure 2).

Early targets of key lupus autoantigens are often restricted and then diversify over time in a concept termed epitope spreading (Arbuckle et al. 1999; James and Harley 1998; McClain et al. 2005). These initial lupus autoantigen humoral targets have proven quite interesting in that immunization of select animal strains with these sequences constructed on a polylysine backbone develop not only antibodies to the peptide of immunization, but also antibodies that bind to the parent protein and eventually develop other autoantibody specificities and clinical features of systemic autoimmunity (McClain et al. 2005). Interestingly, initial targets of Sm B′ and 60kD Ro are cross-reactive with sequential regions of EBV nuclear antigen-1 (EBNA-1), the key latent protein of EBV. This work suggests potential pathways for molecular mimicry to lead to subsets of lupus.

Using new sensitive ELISA assays, studies of pediatric and adult lupus patients and healthy controls drawn from relatives or a large pedigree study have shown an association of EBV seroconversion and SLE [reviewed by James et al. (2006); McClain et al. (2001); Poole et al. (2006)]. In addition, several studies also evaluated the presence of EBV DNA in peripheral blood mononuclear cells and showed an association between EBV DNA presence and lupus (James et al. 1997; Lu JJ et al. 2007; Moon et al. 2004; Yu et al. 2005). Lupus patients had a 15-fold (Moon et al. 2004) to 30-fold (Kang et al. 2004) increase in EBV DNA in the peripheral blood compared with controls; however, no difference in EBV DNA levels was found in mouthwash samples (Moon et al. 2004). The controls in these studies were described as healthy and were matched by demographic factors, but the recruitment process was not described in detail.

More recent studies using isolated B cells from lupus patients and controls showed a 10-fold increase in infected cells as well as differences in EBV gene expression (Gross et al. 2005). These increased levels of EBV infection and gene expression in lupus patients could lead to *a*) a stronger or altered immune response to EBV proteins with resultant cross-reactive self immune responses, *b*) an increased activation state of the host immune response, *c*) a proinflammatory cytokine environment, which could result in easier breaks in tolerance, or *d*) potential EBV-infected autoreactive cells, which could lead to autoantibody formation and/or pathogenic responses.

The host immune response to EBV is also different in lupus patients compared with lupus-unaffected controls. Lupus patients have higher numbers of CD4^+^ T cells but lower numbers of CD8^+^ T cells, which produce interferon-α in response to EBV (Kang et al. 2004). These abnormal responses could contribute to the changes in EBV load seen in lupus patients or provide additional help for abnormal B-cell responses. Antibody responses to EBV are also different. Lupus patients have antibodies against a broad spectrum of early diffuse EBV proteins (Ngou et al. 1992) as well as a higher frequency of antibodies to a larger number of latent nuclear antigens such as EBNA-2 and EBNA-3 (Kitagawa et al. 1988; Lennette et al. 1993). At least two groups to date have shown higher IgA responses in lupus patient sera (Chen CJ et al. 2005; Parks et al. 2005). One of these studies involved a comparison group recruited through a population-based sampling procedure (Parks et al. 2005). SLE reactivities are most similar to patients with chronic viral reactivation. Interestingly, pediatric lupus patients have a broader humoral immune response against EBNA-1 with a larger number of specific humoral epitopes. Areas of reactivity outside of the commonly targeted glycine-alanine repeat are cross-reactive with common early epitopes of self-antigens and are potential targets of molecular mimicry (McClain et al. 2006). Other groups have also found SLE unique humoral immune responses to EBNA-1 (Rhodes et al. 1985).

A variety of bystander (or adjuvant) effects as outlined above for different mechanisms could also be quite important, serving as key links between EBV, and potentially other pathogens, and lupus (Poole and James 2007; Poole et al. 2006). EBV is known to act through different TLRs, which could lead to interferon production, abnormal self-antigen presentation, T-cell activation, cytokine production, and loss of tolerance. EBV has a viral interleukin-10 homolog, which could induce inappropriate APC activation, as well as a bcl-2–like homolog, which can inhibit apoptosis of infected cells. Potential roles for these bystander effects are outlined in Figure 2. Additional evaluation of unique SLE-specific bystander responses to pathogens are warranted.

### Silica: modeling overlapping pathologies for mechanistic clues

Inhalation of silica is associated with overlapping pathologies of inflammation, fibrosis, and autoimmunity. Critical genetic risk factors that confer either susceptibility or, perhaps more intriguingly, protection from silicate-induced autoimmune changes, have not been identified, but clues to early events in autoimmunity may come from studies of the pulmonary effects of silica and the immune dysfunction related to fibrosis (Huaux 2007; Pernis 2005). Mouse strains with different susceptibilities to fibrosis and inflammation make them valuable for teasing apart early processes related to macrophage activation, cytokines, and gene regulation related to silica (Barbarin et al. 2005; Migliaccio et al. 2005; Misson et al. 2004).

New Zealand mixed (NZM) mice showing a mild lupus-prone phenotype (Rudofsky and Lawrence 1999) exhibit exacerbation of lupus pathology after intratracheal silica exposure (Brown et al. 2003). Survival in silica-exposed NZM mice was decreased, with increased proteinurea levels and immunoglobulin (Ig) G deposition suggesting exacerbated kidney damage. The lungs in these mice also had increased inflammatory infiltrates and fibrotic lesions that were well established after 14 weeks, concurrent with significant elevation of autoantibody levels (Brown et al. 2003). Although there are limited data in mice regarding their chronological relationship, there is some evidence that autoantibodies precede and contribute to fibrotic changes in humans [reviewed by Jindal and Agarwal (2005)].

**Figure 3:**
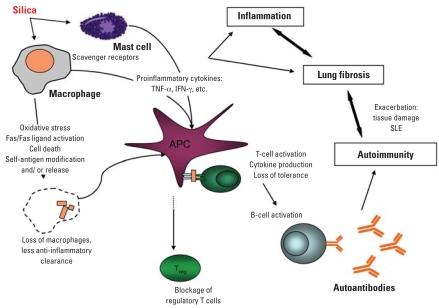
Possible immune-related effects of silica in relation to SLE pathogenesis. Apoptosis of macrophages needed for efficient clearance of debris, along with proinflammatory cytokines, results in uptake of apoptotic debris by activated APCs. The self-antigen may be altered structurally or spatially by oxidative stress. Inflammation helps drive both fibrosis and autoimmunity, resulting in reciprocal exacerbation.

It has been hypothesized that these overlapping outcomes may be related at the level of innate immune responses (adjuvant effect) to silica, possibly via scavenger receptors such as SR-A and MARCO on macrophages and mast cells (Brown et al. 2007; Hamilton et al. 2006). Mice lacking either SR-A or MARCO show different responses to silica versus titanium dioxide, which causes inflammation but not fibrosis or autoimmunity. In addition, there are striking differences in expression of these receptors and silica uptake by cells from Balb/c and C57Bl/6 mice (Hamilton et al. 2006), offering strain and receptor explanations for differential fibrotic susceptibility. The role of scavenger receptors as proinflammatory pathways is complicated by their additional roles in apoptosis induction and the clearance of apoptotic debris. A possible unifying model implicates silica-induced apoptosis of the very cells needed for apoptotic clearance, in an environment of silica-enhanced antigen presentation by dendritic cells or alternately activated macrophages (Beamer and Holian 2007; Migliaccio et al. 2005) (Figure 3), similar to the autologous macrophage killing caused by demethylated T lymphocytes (Richardson 2007).

The silica-induced exacerbation of lupus pathology in NZM mice was ameliorated in mice coinstilled with rottlerin, a putative PKC-δ inhibitor (Brown et al. 2005). Although rottlerin can have PKC-δ–independent effects (Kurosu et al. 2007), as a PKC-δ inhibitor, it is antiapoptotic (Shukla et al. 2003). Further supporting the role of apoptosis in the exacerbation of the NZM lupus model, autoantibodies in silica-exposed mice were shown to bind to macrophages undergoing apoptosis (Pfau et al. 2004). In addition, the Fas/Fas ligand system is up-regulated with silica exposure in both humans and rodents, which could affect its autoimmune effects by increasing apoptosis (Delgado et al. 2006; Otsuki et al. 2006). Because silica can cause oxidative stress and apoptosis in macrophages (Hamilton et al. 1996; Hu et al. 2006), it is possible that these events lead to clustering or proteolytic cleavage of autoantigens. Studies have shown that silica can lead to altered proteosomal processing of specific scleroderma autoantigens (Chen and von Mikecz 2005; Chen M et al. 2005) and activation of apoptotic pathways involving various caspases (Brown et al. 2004a).

Much of the recent data regarding the effects of silica on lymphocyte populations in mouse models of lupus were recently reviewed (Brown et al. 2004b). In silica-exposed NZM mice, lymph nodes had local reduction in regulatory T cells despite a dramatic increase in CD4^+^ T cells (Brown et al. 2004a). Wu et al. (2006) have shown that the function of regulatory T cells is reduced in silicosis patients. Recently, Carlsten et al. (2007) evaluated several serologic measurements for their potential as early markers of immunologic effects of occupational silica exposure in 11 men and found a reduction of CD25^+^ T cells and some increases in T-helper cell (Th)1 and Th2 serum cytokines. However, the study design did not distinguish the CD25^+^ cells as activated versus regulatory T cells. In NZM mice, a relative reduction in serum IgG1, along with elevated tumor necrosis factor-α in lung lavage, suggested a possible Th1 skewing by silica (Brown et al. 2004a), consistent with studies in rats and non-lupus-prone mice (Davis et al. 2001; Garn et al. 2000). Although the roles of tumor necrosis factor-α and Th1/Th2 cytokines in lupus remain unclear (Gomez et al. 2004; Singh 2003), reported increases of interferon-γ with silica are consistent with development of lupus (Carlsten et al. 2007; Garn et al. 2000).

Clearly, silica affects the immune system at several levels that could play roles in lupus pathogenesis. Figure 3 summarizes these studies into a hypothetical model in which no single pathway is causative in itself, but various chronological or simultaneous combinations ultimately result in overt disease.

### Trichloroethylene effects in a lupus mouse model

The female MRL+/+ mouse develops a lupuslike disease late in life (50% mortality at 17 months) and has been used in a series of studies of trichloroethylene. Short-term intraperitoneal trichloroethylene exposure (10 mmol/kg every 4 days for 6 weeks) resulted in increased spleen weight, as well as some serum markers of systemic autoimmunity including total IgG and antinuclear antibodies (Khan et al. 1995). A chronic oral exposure study (21, 100, or 400 mg/kg/day trichloroethylene in drinking water for 32 weeks) also resulted in an accelerated autoimmune response, with increased antinuclear antibodies after 4 weeks of exposure to concentrations as low as 21 mg/kg/day (Griffin et al. 2000b). Chronic treatment with trichloroethylene did not accelerate the development of the lupus nephritis, but after 32 weeks of exposure, tissue pathology commensurate with autoimmune hepatitis was seen. In both mice and humans the majority of trichloroethylene absorbed into the circulation is metabolized by an oxidative pathway in the liver (Lipscomb et al. 1996), converting trichloroethylene to trichloroacetaldehyde, which in solution is in equilibrium with trichloroacetaldehyde hydrate. Female MRL+/+ mice treated for 40 weeks with drinking water containing concentrations of trichloroacetaldehyde hydrate that encompassed the molar equivalents of previous low-level trichloroethylene exposure did not develop autoimmune hepatitis or lupus nephritis but did develop a dose-dependent alopecia and skin inflammation (Blossom et al. 2007).

Trichloroethylene and trichloroacetaldehyde hydrate were also shown in these experiments to increase percentages of activated interferon-γ producing CD4^+^ T cells (Blossom et al. 2004; Griffin et al., 2000b). A recent study that reported higher levels of interleukin-2 and interferon-γ levels in 35 trichloroethylene-exposed workers (mean exposure levels 35 mg/m^3^) compared with 70 nonexposed workers (Iavicoli et al. 2005) provides evidence of similar early immune responses in humans and the MRL+/+ mouse model.

During its metabolism, some trichloroethylene is converted to a trichloroethylene oxide reactive intermediate, which may ultimately lead to the formation of N^6^-formyl lysine or N^6^-dichloroacetyllysine adducts. These adducts have been detected as stable neoantigens in the liver of trichloroethylene-treated MRL+/+ mice (Griffin et al. 2000a), and adduct-specific antibodies have been detected in trichloroethylene-treated MRL+/+ mice (Halmes et al. 1996). Trichloroethylene treatment also promoted the development of antibodies specific for unmodified liver microsomal proteins (Gilbert et al. 2006). Thus, it appeared that trichloroethylene exposure could trigger an immune response against both unmodified and trichloroethylene modified liver proteins. Trichloroethylene may also perturb the immune system through the induction of oxidative stress, as seen by the increased serum levels of inducible nitric oxide synthase (iNOS) and nitrotyrosine in a chronic duration (48 weeks) drinking-water exposure study in female MRL+/+ mice (Wang et al. 2007). Antibodies against lipid peroxidation-derived aldehydes malondialdehyde and 4-hydroxynonenal were also seen. At least some of the oxidative stress generated by trichloroethylene occurred in the liver (Gilbert et al. 2006). Although trichloroethylene-induced adducts and oxidative stress can be immunogenic, the role of these altered self-antigens and the resulting antibodies in disease pathology remains to be determined.

T-cell resistance to activation-induced apoptosis has been seen in patients with lupus, alopecia, and scleroderma (Luzina et al. 2003; Xu et al. 2004; Zoller et al. 2004) and was also seen in these studies of trichloroethylene-exposed mice. Almost 88% of the activated CD4^+^ T cells isolated from control MRL+/+ mice at the 4-week time period were induced to undergo activation-induced apoptosis *in vitro*. In contrast, only 57% of CD4^+^ T cells from mice exposed for 4 weeks to trichloroacetaldehyde hydrate underwent apoptosis. This effect was subsequently linked to a decrease in FasL expression on the CD4^+^ T cells (Blossom and Gilbert 2006). A trichloroacetaldehyde hydrate–induced down-regulation of FasL could enable activated self-reactive CD4^+^ T cells to escape Fas-mediated deletion but retain effector function.

Gilbert et al. developed a model to synthesize the results from these experiments [see Figure 3 in Gilbert et al. (2006)]. Metabolism of ingested trichloroethylene leads to the generation of adducts on liver proteins such as CYP2E1. Trichloroethylene also induces oxidative/nitrosative stress in the liver. The damaged liver cells expressing chemically modified antigens may be taken up by phagocytic cells such as Kupffer cells or hepatic stellate cells. Chemokines secreted by the phagocytic cells help recruit CD4^+^ T cells, which are then presented with unmodified and/or modified liver antigens. Normally, liver-specific CD4^+^ T cells would be deleted by activation-induced apoptosis before they mediated pathology. However, trichloroethylene works via metabolite trichloroacetaldehyde hydrate to downregulate expression of FasL on the CD4^+^ T cells, thereby decreasing their susceptibility to Fas-mediated apoptosis. This effect increases longevity of liver-specific CD4^+^ T cells and thus promotes liver damage commensurate with autoimmune hepatitis. Mice treated with trichloroacetaldehyde hydrate directly do not undergo hepatic adduct formation and/or oxidative stress. Consequently, trichloroacetaldehyde hydrate–mediated inhibition of activation-induced T-cell apoptosis does not manifest itself as hepatitis. The reason why the inflammatory disease is instead directed to the skin and the applicability of this model to specific autoimmune diseases in humans (including lupus, scleroderma, and autoimmune liver disease) remain to be determined.

## Research Gaps and Recommendations

Federal agencies and private foundations have organized several meetings in recent years concerning many aspects of lupus, including the sex and ethnic disparities and prospects for development of new treatments. The goal of one workshop, “Lupus & the Environment: Disease Development, Progression and Flares” (held 8–9 September 2005 in Washington, DC), was to appraise the state of the science and produce recommendations for new research to better understand environmental influences and gene–environment interactions in lupus. The workshop produced a prioritized list of recommendations for research support. Similar issues are described in *Future Directions of Lupus Research*, a recent publication from the National Institute of Arthritis and Musculoskeletal Diseases (NIAMS 2007). Progress has been made and some interesting research has been published since this workshop, but the recommendations that came out of the workshop continue to apply:

- Continued development and increased use of improved lupus-prone and non-prone animal models that are appropriate for research on mechanisms linking environmental exposures to lupus. Models with varying degrees of penetrance and varying manifestations of disease are needed.
- Identification of molecular or physiologic targets of exposures leading to either incidence or progression of lupus, building on studies of emerging concepts such as epigenetics, post-translational steps, metabolic mechanisms, understanding bystander/adjuvant effects of exposure, investigation into “inappropriate” autoimmune responses to common exposures, and multiple exposure studies that test synergy of various factors or agents.
- Development and dissemination of improved technologies and instrumentation to assess environmental exposures integrated over the relevant etiologic time period, including geocoding methods for use with geographic information systems (Nuckols et al. 2004) and biosensors (Schwartz and Collins 2007), techniques involving specific biomarkers of exposure, and questionnaire- or interview-based derivation of specific exposure histories (Parks and Cooper 2006).
- Multisite collaborations with standardized protocols for collection of environmental exposure data focusing on the period before development of clinically expressed disease. These large-scale studies are needed to address the considerable heterogeneity among lupus patients in genotypic profile, in serologic and phenotypic expression, and potentially in etiologic pathways.
- Further study of gene–environment interactions in both human and animal settings. Of particular interest is the identification of lupus-specific versus more general autoimmune disease genes and the exposures that trigger flares for particular genomic profiles.

Our understanding of the mechanisms involved in the pathogenesis of lupus continues to expand. This understanding provides the opportunity to begin to assess whether and how environmental exposures contribute to this process. Clearly the specific environmental exposures discussed in this review are unlikely explanations for the extreme disparity in disease rates seen among women and among ethnic minorities. It will take much more work from a variety of disciplines to address these issues. We believe that acting on these recommendations will enhance our ability to design research studies that address both how and why the pathology of lupus arises, so the devastating impact of this disease can be ameliorated.
